# Children, Disasters, and Place Attachment: A Contemporary Framework for Understanding Crisis in Context

**DOI:** 10.1007/s11920-025-01634-4

**Published:** 2025-08-19

**Authors:** Amethyst Freibott-Kalt, Xin Jiang, Ashley Rose, Joshua Cathcart, Emily-Marie Pacheco

**Affiliations:** 1 Clinical Psychology, Health in Social Science, Old Medical School, University of Edinburgh, Teviot Pl, Edinburgh, EH8 9AG UK; 2 Salvation Army Centre for Addiction Services and Research, Faculty of Social Sciences, University of Stirling, Stirling, FK9 4LA UK

**Keywords:** Place attachment, Children, Disaster, Recovery, Risk, Framework

## Abstract

**Purpose of Review:**

This article critically examines the disaster literature from the past three years (2022–2025) to evaluate the relationship between place attachment and children’s experience of disaster response and recovery.

**Recent Findings:**

Place attachment offers a systematic lens through which we comprehensively map our understanding of the factors that shape, and are shaped by, lived experience of disaster amongst children. We outline why specific consideration of children’s health and wellbeing is significant through this lens, and further consider place attachment in relation to factors identified across relevant bodies of literature. Findings are synthesized across three interdependent, cyclical dimensions: (1) disaster context, including type, location, infrastructure, and planning, (2) children’s holistic experiences of place attachment, including emotional, physical, cultural, and identity-based connections; and (3) disaster outcomes such as displacement, recovery, and rebuilding.

**Summary:**

We propose suggestions for future research, particularly emphasizing the need for an expanded evidence-based, conceptual framework that builds on the model presented in this paper.

**Clinical trial number:**

Not applicable.

I wish that all the mud and debris is washed away from our village as soon as possible; I wish that schools open very soon; I like to see my friends as soon as possible and play with them. [1].

## Introduction

This paper presents a critical review of the disaster literature between 2022 and 2025 to evaluate the relationship between place attachment and children’s experience of disaster response and recovery. Disasters are impacting an increasing number of children worldwide, defined as individuals under the age of 18 [2]. Nearly half of all children are at ‘extremely high risk’ of experiencing disaster due to climate change impacts [3]; and more than 473 million children live in conflict-affected zones [4]. Focusing on the role of place attachment in children’s disaster response and recovery, we argue for an expanded, intersectional framework that considers how children’s psycho-social ties to places shape, and are shaped by, their experiences of hazards and disasters. We contribute an evidence-based adaptation to traditional place attachment theory, that sees traditionally independent categories (e.g., person, place, process) applied as interdependent and dynamic factors that cyclically shape our larger phenomena of interest (i.e., disaster experienced by children). Our conceptual, structured review builds on evidence across related bodies of literature (in disaster, resilience; children’s health, wellbeing, and psychology, etc.) and is intended to inform theory and practice in disaster risk management and safeguarding children’s wellbeing in disaster contexts. We also contribute suggestions for future research aimed at furthering our understanding of active mechanisms for children experiencing hazard events, and present a synthesized model to support responsive, equitable, and developmentally attuned interventions for children experiencing disaster.

## Framework

### Defining Disasters

*Disasters* are events that occur when hazards (whether natural, human-induced or a combination of both) interact with pre-existing risk factors (vulnerabilities), resulting in economic, material, environmental, and human loss [5]. The term thus refers to the human experience, or social consequence, of hazard events; focus on the human, psychosocial experience, is an important distinction when considering the impact of naturally-occurring hazards as we consider these events as *disasters* when intersecting with vulnerabilities and causing significant harm to human systems [6]. While most prior research has framed disasters as linear processes encompassing pre-, peri-, and post-disaster phases (e.g [7]), recent literature has begun to conceptualize disasters as a continuum in which numerous interdependent domains influence our understanding, management, and experiences of disasters [8]. In particular, longitudinal research evidences the dynamic and reciprocal nature of interactions between disaster outcomes and the broader socio-ecological contexts in which they occur [9, 10]. Therefore, we conceptualize disasters as evolving, nonlinear processes of social consequence shaped by interdependent social-ecological domains, unfolding across time and space.

## Understanding Disaster Impacts on Children Through Place Attachment

Scannell and Gifford [11] define place attachment as a multidimensional psychological bond between people and places, structured across three dimensions: person, process, and place (PPP). *Person* refers to who is attached; *process* captures emotional, cognitive, and behavioral mechanisms; and *place* concerns the physical and social characteristics of the location. Within the psychological literature, this tripartite framework positions place attachment as a dynamic relationship shaped by both individual experience and environmental context. Similarly, the term *socio-ecological* is used in disaster resilience literature [12–14] to describe place attachment dynamics because it captures the interconnectedness of people (social) and their environments (ecological), both of which are central to how individuals and communities experience, respond to, and recover from disasters (see also [15]). We draw on vocabulary from both domains to present accurate and integrated insights that are accessible to a broad range of stakeholders. However, we identify ‘place’ as the primary intersecting focus, serving as a cross-cutting dimension of interest within both psychological and disaster research.

As the context in which we live our lives, *place*, functions as a locus of meaning that shapes perception, identity, and social experience [16]. For children, place plays a particularly formative role, providing the environmental and relational grounding through which emotional and social bonds are established and through which the world is interpreted [17]. Children are especially vulnerable in disaster settings due to their developmental stage and dependence on adults for survival [18–21]. Research highlights the role of place dynamics in fostering a sense of identity, safety, stability, belonging, and social grounding [22, 23]. Such attachments are often influenced by factors such as environmental stability, sensory characteristics, and socially embedded meanings within physical settings.

Disasters present acute disruptions to place, with complex consequences mediated by a range of biopsychosocial processes, including collective trauma and loss. These disruptions may result in emotional disorientation and rupture, but in some cases can also promote resilience through the reconstruction of place-based identities [23–25]. To further explain, stable place environments are associated with positive developmental outcomes, including psychological well-being, cognitive development, and motor and social functioning [23, 26]. However, disruptions to significant places can negatively affect multiple domains of development. For example, the loss of home and school settings has been linked to declines in educational engagement and emotional regulation [27], while separation from familiar neighborhoods and peer networks can lead to increased anxiety and behavioral challenges [21]. Additionally, disruptions to culturally meaningful places (such as community gathering sites or religious spaces) may result in a loss of identity continuity and reduced social integration [28]. Few studies have directly examined children’s experiences of disasters, and fewer have explored how disrupted place attachment contributes to children’s risk and recovery trajectories.

A place attachment lens offers a valuable framework for exploring these complex dynamics in children’s experiences. Advancing our understanding in this area is essential for enhancing disaster preparedness and supporting resilient recovery efforts, especially given the current lack of conceptual attention to children’s place-based experiences in disaster contexts. We subsequently expand on the evidence-base informing our position, structured as a synthesized model.

## Cyclical Model of Place Attachment for Children in Disaster Settings

**Fig. 1:**
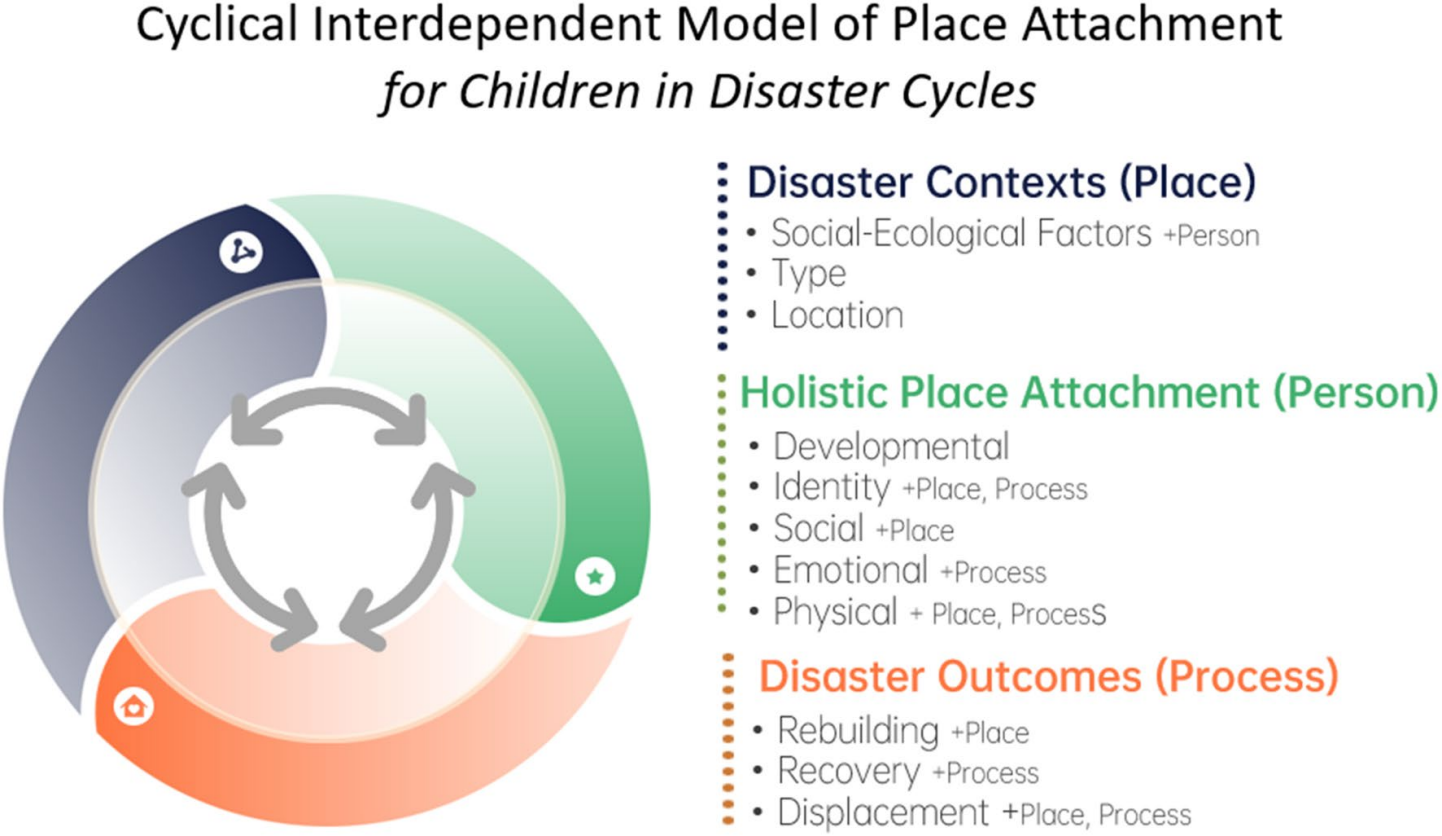
Cyclical model of place attachment for children in the continuum of disaster. Note Place Attachment traditionally comprises three distinct domains of consideration as person, place, and process [11]; we note (not exhaustive) key relations to these domains in brackets and indicate secondary relations with sub-domains via ‘+’. Here, ‘cyclical’ should not be conflated with temporal linearity: despite the parallels with conceptualizations of disasters as temporal repetitive phases (pre-, peri-, post-, repeat), the cyclical nature of the current model rather reflects the reality that context, attachment, and outcomes often involve cascading effects. Internal arrows reflect the bi-directional and interdependent nature of these dimensions, meaning each is known to impact the other (e.g., as cause and consequence), over time

We introduce a conceptual model to critically synthesize evidence-based insights on the role of place attachment in children’s disaster response and recovery. This model recognizes the cyclical nature of disasters, and the interplay between place attachment dimensions and socio-ecological conceptualizations of risk and protective factors (see *Framework)*. Insights are presented as three interdependent dimensions: (1) *disaster context*, (2) *children’s holistic experiences of place attachment*, and (3) *disaster outcomes* (see Fig. 1). These three dimensions majorly align with Scannell & Gifford’s [11] conceptualizations of place, person, and process respectively; but, elements of each (PPP) are present across our model. This is in-line with the notion that the person-place-process domains are inherently interdependent and that real-world experiences often blur these conceptual boundaries [11, 29].

## Disaster Contexts (Place)

### Role of Social-Ecological Factors

As previously explained, disasters are experienced as human responses to the adverse effects of hazards across social, environmental, cultural, and psychological systems. The ways in which individuals experience and understand disaster events are shaped by intersecting factors such as age, race, ethnicity, gender, spirituality, socioeconomic status, displaced status, class, and ability [3, 30, 32, 33]. For children, these social factors are embedded within their lived environments and significantly shape the nature of their place attachments. These attachments, formed through socio-ecological ties, influence psychosocial outcomes when those places are disrupted at any phases of the disaster continuum [32, 34, 35]. For example, the children of the Dene Tha’ First Nation illustrate how land-based identity, spirituality, and well-being are intimately connected through affective, cognitive, and behavioral ties to ancestral land ([12; see also [36]). As climate change degrades their sacred environment, children experience climate anxiety and a growing emotional disconnect. Social identity theory provides a relevant framework, suggesting that collective cultural beliefs are internalized at the individual level [23, 37, 38], influencing children’s capacity for psychosocial regulation when they are affirmed or threatened [39–41]. In such cases, cultural, religious, or group-based representations of value (such as sacredness) serve as intergenerational pathways through which disaster exposure can intensify vulnerability [42–44].

The factors influencing place attachment vary across social and geographic contexts. Children frequently describe disaster experiences in terms of specific places that supported their social relationships and development (e.g., homes, schools, or community spaces). We have already demonstrated how such sites of connection contribute to cognitive, social, and emotional growth. However, the social representations children hold of these places can shape disaster risk and recovery outcomes in both adaptive and maladaptive ways [25]. For example, in a study of post-flood recovery in rural Iran, children expressed ‘faith’ in the interconnectedness of their community and a shared determination to rebuild valued spaces [1]. These spaces, which enabled social engagement among peers, were seen as integral to their recovery. The literature supports this notion, suggesting that such environments are central to identity development, offering opportunities for self-discovery which are embedded positively in their personal memories [43, 45]. Disaster recovery efforts often prioritize rebuilding of homes, schools, and communal settings as they provide structured opportunities for interaction with peers and community members, reinforcing social identity and belonging [17, 46]. Yet, strong attachments to these places may lead to reluctance to relocate following disaster. While social support networks and future-oriented planning are recognized protective factors for mental well-being, children’s resistance to relocation, motivated by their place attachments, can lead to underestimation of risk [25] and hinder disaster risk management initiatives that rely on evacuation or resettlement [47]. As such, positive social representations of place as ‘home’ or ‘valued’ may function as both protective and risk factors.

### Role of Disaster Type, Location, and Structural Inequality

Disaster risk management must be grounded in local realities. When applied across divergent cultural and environmental contexts, standardized approaches may overlook how place is experienced and understood [47]. Most children globally live in the Majority World, where disasters are often slow onset and deeply embedded in daily life [48, 49]. Climate change, for instance, gradually erodes physical and social environments, disrupting routines and limiting access to spaces that support identity and cohesion [17, 31].

Understanding locally held meanings of homes, schools, and neighbourhoods can clarify how development, vulnerability, and attachment intersect in disaster contexts [12]. In contrast to prevention-oriented approaches in the Minority World [50], rural Majority World settings often lack resources for disaster preparedness [18]. In rural Iran, for instance, children described widespread destruction and community fragmentation following floods in a context unprepared for large-scale disaster [1].

Such environments are particularly vulnerable due to limited infrastructure, institutional capacity, and prolonged recovery timelines [25]. In slow onset disasters like climate change, place attachments are weakened over time, while opportunities to develop new ones remain constrained [12]. Displacement exacerbates these challenges, disrupting identity development and mental well-being [23, 24]. Rahmani and colleagues [31] found that youth suicidality surged one year after disasters in rural areas, underlining the consequences of prolonged disruption to social and environmental continuity.

## Role of Children’s Voices and Individual Meaning-Making

Allowing children to speak on behalf of themselves is critical for effective disaster risk management. Adult-driven social representations of place often fail to capture the complexity of how children understand and respond to disruption [17, 51]. For children, these understandings are formed at the person level, through their direct experiences of place and relationships within it. While group level representations (such as shared cultural or national identities) shape collective experiences of place, children’s attachments often reflect intimate, lived relationships with specific environments [52]. These person-level meanings are not isolated, but emerge in interaction with wider social, cultural, and ecological contexts. Recognising this complexity is crucial for understanding how place attachments form and how they influence risk perception and recovery for children in disaster contexts.

Thus far, we have examined children’s place attachments primarily through social ecological contexts and their embeddedness in physical and social environments. We now shift the focus to *person* as the primary domain of place attachment, with secondary attention to *place* and *process*. Specifically, we examine how children’s holistic attachments shape and are shaped by their disaster experiences. In line with traditional place attachment models (e.g [17]). we conceptualize place not only as a physical setting but also as a social arena, through which children’s attachments emerge via ongoing interaction and meaning-making.

## Holistic Experiences of Place Attachment (Person)

Children’s place attachments are developed through holistic experiences that integrate personal, social, and physical dimensions of place. As Grimshaw and Mates [53] argue, place serves as a site for emotional, physical, and social meaning-making. Children interact with these environments in developmentally specific ways: while younger children often focus on functional use, adolescents tend to emphasize symbolic and social meanings [54]. Across age groups, personal memories, embodied interactions, and subjective experiences form the foundation of attachment, contributing to children’s sense of stability, identity, and well-being (e.g [55]). Disasters introduce new forms of place-based memories, which can alter children’s ongoing relationships with their trusted and valued environments.

Personal engagements with place become particularly salient in disaster contexts, where affective meaning is often intensified. Children’s sensitivity to the emotional significance of disrupted environments can reveal interpretive details that may be overlooked by adults. For example, Parrott and colleagues [46] found that adolescent survivors of a multi-hazard disaster (flood, tsunami, landslide) in rural Indonesia described specific micro-sites within their school that held traumatic meaning- such as the cafeteria, where a lifeless body was found. Similarly, Sadeghloo and Mikhak [1] report that material damage to community spaces following disaster frequently evokes memories of social rupture, linking physical destruction to emotional loss. Such sense of loss, including the loss of trust and sense of safety in a valued environment, is particularly difficult for children (in post-disaster contexts) to psychologically regulate [56, 57, 58]. Such findings illustrate how children’s internal meaning-making processes interact with disrupted external environments, and how place-based memories can further alter children’s relationships with various environments and their social representations. Put simply, these bi-directional person-based processes contribute to recovery trajectories and inform how children relate to place in post-disaster contexts.

This relationship between children’s internal affective processes and their engagement with social and physical environments is particularly evident in school settings. Schools are often central to children’s social development, offering routine, structure, and peer interaction. In the aftermath of disasters, they may facilitate community recovery by restoring social connection, emotional safety, and group-based coping activities [46, 59]. Yet, schools can also become sites of risk, representing disrupted social ecologies, broken routines, or physical danger. These dual representations underscore the complex interplay between individual experiences and environmental context in children’s disaster recovery.

Beyond formal institutions, children also form attachments to informal and personal environments. The concept of *found places* encompasses small, self-selected settings (such as alleyways, footpaths, or boulders), illustrating the role of agency in children’s disaster recovery. These spaces often provide displaced children with a sense of ownership, safety, creativity, and social connection [15]. For example [24], describes how displaced children from an Indigenous group in Nepal formed strong emotional ties to a boulder overlooking their resettlement camp, describing it as the one space that “belonged” to them. Such places often become sites for peer gatherings and shared activities, fostering resilience through social interaction [23]. These examples further support the importance of recognizing children’s individual meaning-making processes and the situated physical and social affordances that support recovery.

In sum, this section highlights how contemporary literature reinforces the understanding that children’s place attachments are grounded in personal emotional processes, shaped by developmental and social needs, and expressed through interactions with both physical and social environments. Recognizing the multi-dimensional nature of these attachments is essential for informing child-centered disaster response and recovery frameworks that account for the complexity of children’s lived experiences. Building on this foundation, the following section shifts focus to *process* as the central domain of analysis, examining the emotional, cognitive, and behavioral mechanisms through which place attachments operate. We consider how these processes unfold within and across the three interconnected dimensions of place attachment and how they shape the outcomes children experience in disaster contexts.

### Disaster Outcomes (Process)

Having explored the *place* and *person* components of place attachment in disaster contexts through a broad social-ecological lens, we now explore the *process* components of disaster experiences for children considering their cognitive, affective, and behavioral responses in both displaced and rebuilding contexts. It is important to note that while research has called for a strengthening of children’s perspectives in the disaster literature [21], the research landscape still suffers from a lack of children’s first-hand disaster accounts. For example, the impact on preschool-aged children is more likely to be looked at through the lens of the mental health of those around them, specifically their caregivers [28]. Therefore, this section will look at disaster outcomes from a children’s place attachment perspective where it has been available in the literature (largely in displacement and post-disaster recovery literature).

### Displacement

Our cyclical framework understands disaster outcomes as continuously evolving over time, reshaping children’s evolving place relationships and future disaster contexts. Research consistently identifies disasters as risk factors that disrupt educational, social [46, 59], infrastructural, cultural, financial [24], and place attachment [17, 23] domains. Disaster recovery is, therefore, not a linear progression but a dynamic restoration of disrupted physical and social environments, unfolding over time and influenced by context-specific factors [60]. Within this framework, children’s place attachments serve as key mechanisms of psychosocial regulation, offering a sense of connection, agency, and stability during recovery [17]. As complex and layered as disaster recovery may be, we conceptualize it as the dynamic, multidimensional restoration of social factors and physical places that evolve over time and are informed by local contexts [59]. Children’s place attachments in disaster recovery play a vital role by providing children with a sense of community connection, social agency, and connection to place [17].

These processual dynamics are especially noteworthy in the context of displaced children. In 2024, UNICEF reported over 3.1 million children have been displaced due to natural disasters, and over 47 million have been displaced due to conflict and violence [61]. Displacement, defined as the forced movement from one’s home due to environmental or human-made disasters [62], can disrupt children’s place attachments and create identity confusion [23]. For example, Morrison [24] found that in displaced contexts, generational divides intensified as a result of youth reconsidering their cultural and familial commitments in favor of their personal ambitions and hope for rebuilding after disaster events. Youth were more likely to seek out youth-specific spaces that supported their shifting social aspirations, such as an increased emphasis on education and employment.

These behavioral shifts are closely tied to spatial processes: micro elements of place (e.g., solar-powered street lights expanding place access and found spaces) become crucial elements of the resettling and recovery process for displaced youth as they rebuild their sense of self and their connection to place. Weir and colleagues [23] further emphasize the effects of displacement on children’s place attachments, noting that displacement often leads children to navigate their sense of self in unfamiliar physical environments. Through social interaction and play, displaced children may take on a sense of ownership of their new location and, in doing so, forge new place attachments that serve as capacity-building factors in post-disaster recovery. These findings are increasingly important as gradual environmental degradation and protracted displacement sever children’s bonds to familiar environments (e.g., homes, playgrounds, and culturally significant sites) triggering profound mourning and heightened anxiety [12, 31].

### Rebuilding

Where relocation is not required, disaster recovery often takes the form of rebuilding, which also engages affective, cognitive, and behavioral processes. Crandon and colleagues [12] apply Bronfenbrenner’s [51] social ecological model to illustrate how attachments to home (microsystem), school (mesosystem), and cultural landscapes (macrosystem) function as buffers against acute and chronic stressors following disaster. Restoring these spaces facilitates emotional and social continuity, such as through reopening schools and playgrounds, or reviving cultural rituals in rebuilt environments. Pacheco and colleagues [46] similarly argue that schools, as familiar and symbolically meaningful places, can become physical markers of recovery. They provide structured environments for restoring routines, reconnecting with peers, and regaining a sense of belonging [59]. The rebuilding of community spaces is often the most effective means of fostering restoration of place ties, which directly aid in children’s recovery [1, 46].

Yet, the processes of rebuilding are not universally positive. Children’s affective and behavioral responses to rebuilding are nuanced and sometimes conflicted. In an extensive review, Carone and colleagues [25] found that individuals with strong place attachments may engage more thoughtfully in rebuilding, while others resist it, perceiving it as a threat to the integrity of their place-based identities. For children, rebuilding familiar environments may represent both recovery and risk. While it can foster a sense of continuity and emotional safety, it may also reintroduce distressing memories or challenge children’s evolving place meanings [17, 46]. These divergent responses highlight the importance of process-oriented understandings of recovery, that are also sensitive to children’s developmental stage, emotional needs, and (of critical importance) sense of agency.

Taken together, this paper’s review of current literature regarding disaster outcomes and children’s place attachments posits that children have far more agency in their own recovery than they are often credited for. By examining disaster outcomes through the process domain of place attachment, we found that supporting children in post-disaster settings should take into account their unique perspectives and apply a holistic understanding of disasters as cyclical events and place attachments as key mechanisms of recovery across physical, social, emotional, and individual domains.

## Conclusion

### Strengths & Limitations

This article synthesized recent literature to provide a nuanced understanding of the importance of place attachment in children’s disaster response and recovery. We offer a broad conceptual framework that may guide future research. To our knowledge, the proposed cyclical relationship between these elements is a novel contribution that supports more intersectional approaches to children’s vulnerability and agency in disaster contexts. A key limitation is the limited representation of children’s voices in the literature, though we prioritized studies that directly examined children’s place attachments and disaster experiences. Included studies ranged from systematic reviews to geographically and culturally specific cases, though potential regional and hazard-type biases remain due to literature availability. Future research should also consider the unique needs of children that require additional support such as neurodivergent children and those with disabilities.

### Recommendations for Future Research

To build on the proposed framework, future research should prioritize longitudinal studies that examine the evolution of children’s place attachments across the disaster continuum. For example, studies should investigate how disaster resilience may be understood *through* or *within* the dynamics of the cyclical model (Fig. 1). This requires age-specific approaches that center children’s perspectives to ensure research is relevant and meaningful. An intersectional, social ecological lens is also essential for exploring how place attachment functions across diverse contexts. As natural and human-made disasters increase and social vulnerabilities limit recovery, comparative studies are needed to examine place attachments in both placed and displaced contexts, within and beyond the Majority World. Further research into the micro and macro mechanisms of place attachment will also enhance our understanding of its role in children’s disaster response and recovery.

Despite a growing body of work, children’s voices remain underrepresented in disaster literature. While many studies stress the importance of including children in recovery processes (e.g., [17 [23, 32, 59]), few offer practical guidance on how to meaningfully involve them. Yet evidence shows that children attribute significance to both symbolic landmarks and micro-level elements such as walls, paths, or rooms. Their sensitivity to both material and symbolic features of place supports their role as active agents in recovery. Future research must explore children’s agency more explicitly to inform inclusive, effective strategies for disaster resilience.

We began this paper with the voice of a child affected by catastrophic flooding in rural Iran; a voice longing for the restoration of safe, meaningful places. As disasters continue to increase globally, more children are losing their homes, communities, and social networks. Now more than ever, child-centered approaches to disaster response and recovery are essential. Applying a place attachment lens to children’s disaster experiences can help guide the development of responsive, equitable, and age-appropriate interventions. Our collective responsibility is to build disaster-resilient societies in which every child has access to places where they feel safe, connected, and a sense of belonging.

## Data Availability

No datasets were generated or analysed during the current study.
